# Memoranda of Anatomy, Surgery and Physiology; Forming a Pocket Companion for the Young Surgeon, or for Students Preparing for Examination

**Published:** 1848-07

**Authors:** 


*Memoranda of Anatomy, Surgery and Physiology; forming a pocket companion for the young Surgeon, or for Students preparing for Examination.* By MARK NOBLE BOWER, Surgeon. Corrected and revised by an American Physician. 18mo., pp. 325. Samuel S. & Wm. Wood. New York, 1848.

This is a concise Summary of the subjects indicated in the title page, and, as far as we have looked into it, a very correct one. From its brevity it is well adapted to refresh the memory, although little calculated to supply all the requisite information upon subjects so important and extensive.

